# Effect of Coating on the Strain Transfer of Optical Fiber Sensors

**DOI:** 10.3390/s110706926

**Published:** 2011-07-01

**Authors:** Shiuh-Chuan Her, Chih-Ying Huang

**Affiliations:** Department of Mechanical Engineering, Yuan Ze University, Chung-Li 32003, Taiwan; E-Mail: s907219@mail.yzu.edu.tw

**Keywords:** optical fiber strain sensor, Mach-Zehnder interferometer, strain transfer, bonded length

## Abstract

Optical fiber strain sensors with light weight, small dimensions and immunity to electromagnetic interference are widely used in structural health monitoring devices. As a sensor, it is expected that the strains between the optical fiber and host structure are the same. However, due to the shear deformation of the protective coating, the optical fiber strain is different from that of host structure. To improve the measurement accuracy, the strain measured by the optical fiber needs to be modified to reflect the influence of the coating. In this investigation, a theoretical model of the strain transferred from the host material to the optical fiber is developed to evaluate the interaction between the host material and coating. The theoretical predictions are validated with a numerical analysis using the finite element method. Experimental tests are performed to reveal the differential strains between the optical fiber strain sensor and test specimen. The Mach-Zehnder interferometric type fiber-optic sensor is adopted to measure the strain. Experimental results show that the strain measured at the optical fiber is lower than the true strain in the test specimen. The percentage of strain in the test specimen actually transferred to the optical fiber is dependent on the bonded length of the optical fiber and the protective coating. The general trend of the strain transformation obtained from both experimental tests and theoretical predictions shows that the longer the bonded length and the stiffer the coating the more strain is transferred to the optical fiber.

## 1. Introduction

The use of optical fiber sensors has grown considerably over the past decade in applications ranging from the aerospace industry to civil engineering [1,2]. Optical fiber sensors have been developed to measure a variety of physical quantities such as strain, temperature and vibration. Smart structures which integrate both sensors and actuators have the ability to sense environmental changes within or around the structure and are able to interpret and react to these changes. Optical fiber sensors possess several advantages over conventional electrical sensors such as light weight, small dimensions, high temperature endurance, dielectric nature, immunity to corrosion, electromagnetic interference and electric hazards, that meet the basic sensing requirements of smart structures to a large extent. The direct application of smart structures in structural health monitoring has been studied intensively over the last few years, particularly in buildings and bridges [3–5]. Several fiber optic technologies have been proposed for strain/temperature measurements, such as fiber Bragg grating (FBG) sensors [6–8], high-polarimetric fiber optic sensors [9,10] and white-light interferometric fiber sensors [11]. Fiber Bragg grating based optical fiber sensors have been demonstrated successfully in monitoring structures. Liu *et al.* [12] used polymer-coated FBG sensors to measure pressure and temperature simultaneously. Chau *et al.* [13] experimentally demonstrated the effectiveness of using the FBG system for structural vibration control. Interferometric type fiber optic sensors have the advantages of high sensitively and high spatial resolution. Therefore, they can be used for damage detection in structural members [14,15]. As a sensor, it is expected that the strains between the optical fiber and host structure are the same. However, due to the existence of the adhesive layer and protective coating, part of the energy is converted into shear deformation. Thus, the strain of the optical fiber is different from that of the host structure. Lau *et al.* [16] developed a simple model to calculate the percentage of strain applied to the host structure actually transferred to the embedded fiber optic sensor. Zhou *et al.* [17] investigated the influence of coating and adhesive on the strain transmission of embedded fiber optic sensors. Most of the existing works that have studied the strain transfer between the optical fiber and host material have focused on the embedded optical fiber. In reality, the surface bonded optical fiber is much easier to use in structural monitoring applications then embedded optical fibers. Ansari and Libo [18] proposed a theoretical model to study the strain transferred from the host material to the surface bonded optical fiber. They assumed that the optical fiber (with coating) was perfectly bonded with the host material. The effect of adhesive layer was neglected. Wan *et al*. [19] investigated the influence of four geometric parameters of the adhesive, side width, top thickness, bonded length and bottom thickness, on the strain of the surface-attached optical fiber. In this investigation, the fiber optic sensor is surface bonding on the host structure. The theoretical model includes the adhesive layer and protective coating. An analytical formula is proposed to evaluate the real strain in the host structure using the surface bonded optical fiber. This formula provides accurate prediction of the strain transformation rate from the host material through the adhesive layer and protective coating to the optical fiber strain sensor. A numerical study is performed to evaluate the influence of the coating and bonded length on the strain transferred from the host structure to the optical fiber. Experimental tests are conducted based on the Mach-Zehnder interferometery to reveal the differential strains between the fiber-optic sensor and host structure.

## 2. Basic Assumptions

**Figure 1.**
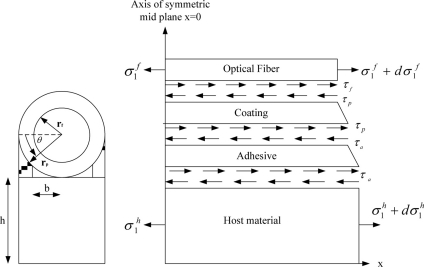
Analytical model of surface bonded optical fiber.

When the optical fiber is used as a sensor to monitor the strain or stress in a structure, the measurement capability depends on the characteristics of the bonding between the optical fiber and host structure. The measurement sensitivity of the fiber optic sensor is affected by the protective coating and bonding length. In this investigation, the optical fiber is surface bonded onto the host structure while an external load (stress or strain) is applied only to the host structure. In fact, the deformation of the optical fiber induced by the host structure is transferred via the adhesive layer and protective coating. The optical fiber deformation induces a change in optical signal transmission. Since the stress or strain is directly applied to the host structure and not the optical fiber, so that the strain or stress in the optical fiber is subjected to shearing at the interface between the fiber and coating. In this presentation, the strain transformation between the host structure and surface bonded optical fiber sensor is derived based on the following assumptions [16,20]:

1. All of the materials including the bare optical fiber, protective coating, adhesive layer and host structure behave as linear elastic isotropic materials.
2. All interfaces are perfectly bonded. Thus, the displacement continuity along interfaces can be described as follows :
  $$u(z)=\{\begin{array}{ll} u_{1}^{h}=u_{1}^{a} & r=r_{a}\\ u_{1}^{a}=u_{1}^{p} & r=r_{p}\\ u_{1}^{p}=u_{1}^{f} & r=r_{f} \end{array}$$
  where $u_{1}^{h}$, $u_{1}^{a}$, $u_{1}^{p}$ and $u_{1}^{f}$ are the displacements of the host material, adhesive, coating and optical fiber, respectively; *r_a_*, *r_p_*, *r_f_* are the radii of adhesive, coating and optical fiber, respectively, as shown in Figure 1.
3. The protective coating and adhesive are subjected to shear deformation only. This assumption is reasonable since the Young’s moduli of the coating and adhesive are significantly smaller than that for the host structure and optical fiber.

## 3. Strain Analysis of Optical Fiber

The analytical model is shown in Figure 1, with a cylindrical optical fiber and coating on the top and the host material at the bottom, and the adhesive in between. The host material is subjected to a far field stress *σ*_0_. Under the assumption (3), the equilibrium equation for the coating is:

(1) $$r_{p}\cdot \int_{0}^{\pi }\tau_{p}(r_{p},\theta ,x)d\theta \cdot dx-r_{f}\cdot \int_{0}^{2\pi }\tau_{p}(r_{f},\theta ,x)d\theta \cdot dx=0$$

where *τ_p_* represents the shear stress in the coating which is inverse to the radius and expressed as:

(2) $$\tau_{p}(r,\theta ,x)=\frac{r_{p}}{r}T(\theta ,x)$$

where *T* (*r*, *θ*), denotes the shear stress at the interface between the coating and adhesive.

The shear strain in the coating is:

(3) $$\gamma_{p}=\frac{\partial u_{1}^{p}(r,\theta ,x)}{\partial r}=\frac{\tau_{p}(r,\theta ,x)}{G_{p}}$$

Substituting Equation (3) into Equation (2), yields:

(4) $$\frac{\partial u_{1}^{p}(r,\theta ,x)}{\partial r}=\frac{1}{G_{p}}\cdot \frac{r_{p}}{r}\cdot T(\theta ,x)$$

where *G_p_* and $u_{1}^{p}$ represent the shear modulus and axial displacement of the coating, respectively.

Integration with respective to radius, gives:

(5) $$u_{1}^{p}(r,\theta ,x)=\frac{r_{p}}{G_{p}}\cdot \text{ln}(r)\cdot T(\theta ,x)+C_{1}$$

Enforcing the displacement continuity in the interface between the coating and optical fiber:

$$u_{1}^{p}(r_{f},\theta ,x)=u_{1}^{f}(r_{f},\theta ,x)$$

The displacement of coating Equation (5) can be rewritten as:

(6) $$u_{1}^{p}(r,\theta ,x)=\frac{r_{p}}{G_{p}}\cdot \text{ln}(\frac{r}{r_{f}})\cdot T(\theta ,x)+u_{1}^{f}(r_{f},\theta ,x)$$

where $u_{1}^{f}(r_{f},\theta ,x)$ is the displacement of optical fiber in the interface between coating and optical fiber.

The displacement continuity in the interface between the coating and adhesive can be written as:

(7) $$u_{1}^{p}(r_{p},\theta ,x)=u_{1}^{a}(r_{p},\theta ,x)=\frac{r_{p}}{G_{p}}\cdot \text{ln}\left(\frac{r_{p}}{r_{f}}\right)\cdot T(\theta ,x)+u_{1}^{f}(r_{f},\theta ,x)$$

The adhesive thickness is angle dependent as shown in Figure 1:

(8) $$t(\theta )=r_{p}-r_{p}\;\sin\theta$$

The shear strain and stress in the adhesive are:

(9) $$\gamma_{a}=\frac{u_{1}^{h}-u_{1}^{a}(r_{p},\theta ,x)}{r_{p}-r_{p}\;\sin\theta };\;\tau_{a}=\frac{u_{1}^{h}-u_{1}^{a}(r_{p},\theta ,x)}{r_{p}-r_{p}\;\sin\theta }\cdot G_{a}$$

Substituting Equation (7) into Equation (9) yields:

(10) $$\tau_{a}=\frac{G_{a}}{r_{p}-r_{p}\;\text{sin}\;\theta }[u_{1}^{h}-\frac{r_{p}}{G_{p}}\text{ln}\frac{r_{p}}{r_{f}}\cdot T(\theta ,x)-u_{1}^{f}]$$

The continuity of the shear stress in the interface between the adhesive and coating leads:

(11) $$T(\theta ,x)=\tau_{a}=\frac{G_{a}}{r_{p}-r_{p}\;\text{sin}\;\theta }[u_{1}^{h}-\frac{r_{p}}{G_{p}}\text{ln}\frac{r_{p}}{r_{f}}\cdot T(\theta ,x)-u_{1}^{f}]$$

Thus, the shear stress *T*(*θ*, *x*) in Equation (11) can be rearranged as:

(12) $$T(\theta ,x)=\frac{1}{\left[\frac{r_{p}(1-\text{sin}\;\theta )}{G_{a}}+\frac{r_{p}}{G_{p}}\text{ln}(\frac{r_{p}}{r_{f}})\right]}\cdot (u_{1}^{h}-u_{1}^{f})$$

Substituting Equation (12) into Equation (1), yields:

(13) $$\int_{0}^{2\pi }\tau_{p}(r_{f},\theta ,x)d\theta =\frac{r_{p}}{r_{f}}\int_{0}^{\pi }T(\theta ,x)d\theta =\frac{r_{p}}{r_{f}}\int_{0}^{\pi }\frac{1}{\left[\frac{r_{p}(1-\text{sin}\;\theta )}{G_{a}}+\frac{r_{p}}{G_{p}}\text{ln}(\frac{r_{p}}{r_{f}})\right]}\cdot (u_{1}^{h}-u_{1}^{f})d\theta$$

The adhesive is filled between the host material and coating with a small gap b as shown in Figure 1. Thus, Equation (13) can be rewritten as:

(14) $$\int_{0}^{2\pi }\tau_{p}(r_{f},\theta ,x)d\theta =\frac{2r_{p}}{r_{f}}\int_{0}^{\cos^{-1}(\frac{b}{r_{p}})}\frac{1}{\left[\frac{r_{p}(1-\sin\theta )}{G_{a}}+\frac{r_{p}}{G_{p}}\ln\frac{r_{p}}{r_{f}}\right]}(u_{1}^{h}-u_{1}^{f})d\theta$$

The equilibrium equation of optical fiber in the x-axis as shown in Figure 1 is:

(15) $$\sigma_{1}^{f}\cdot \pi \cdot r_{f}^{2}=(\sigma_{1}^{f}+d\sigma_{1}^{f})\cdot \pi \cdot r_{f}^{2}+r_{f}\cdot \left[\int_{0}^{2\pi }\tau_{p}(r_{f},\theta ,x)d\theta \right]\cdot dx$$

Now, we differentiate Equation (15) with respect to x twice and incorporate into Equation (14), which leads to:

(16) $$\frac{d^{2}\sigma_{1}^{f}}{dx^{2}}+\frac{2r_{p}}{\pi r_{f}^{2}}(\frac{\sigma_{1}^{h}}{E_{h}}-\frac{\sigma_{1}^{f}}{E_{f}})\int_{0}^{{\text{cos}}^{-1}(\frac{b}{r_{p}})}\frac{1}{\frac{r_{p}(1-\text{sin }\theta )}{G_{a}}+\frac{r_{p}}{G_{p}}\text{ln}(\frac{r_{p}}{r_{f}})}d\theta =0$$

where $\sigma_{1}^{f}$, $\sigma_{1}^{h}$ are the longitudinal normal stresses in the optical fiber and host material, respectively; *E_f_*, and *E_h_* are the Young’s moduli of optical fiber and host material, respectively.

**Figure 2.**
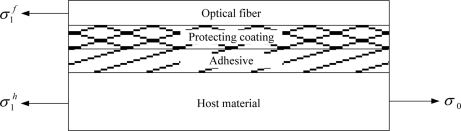
Force equilibrium.

The equilibrium equation of the four materials system as shown in Figure 2 is:

(17) $$\sigma_{1}^{h}=\sigma_{0}-\frac{\pi r_{f}^{2}}{2\cdot h\cdot r_{p}}\sigma_{1}^{f}$$

Substituting Equation (17) into Equation (16), yields:

(18) $$\begin{array}{l} \frac{d^{2}\sigma_{1}^{f}}{dx^{2}}-\lambda_{1}^{2}\sigma_{1}^{f}=-\frac{2r_{p}{ɛ}_{0}}{\pi r_{f}^{2}}\int_{0}^{{\text{cos}}^{-1}(\frac{b}{r_{p}})}\frac{1}{\frac{r_{p}(1-\text{sin}\;\theta )}{G_{a}}+\frac{r_{p}}{G_{p}}\text{ln}(\frac{r_{p}}{r_{f}})}d\theta \\ \;\;\;\;\lambda_{1}=\sqrt{[\frac{2r_{p}}{\pi r_{f}^{2}}(\frac{\pi r_{f}^{2}}{2hr_{p}E_{h}}+\frac{1}{E_{f}})\int_{0}^{{\text{cos}}^{-1}(\frac{b}{r_{p}})}\frac{1}{\frac{r_{p}(1-\text{sin}\;\theta )}{G_{a}}+\frac{r_{p}}{G_{p}}\text{ln}(\frac{r_{p}}{r_{f}})}d\theta ]} \end{array}$$

The solution of differential Equation (18) can be expressed as:

(19) $$\sigma_{1}^{f}=A\;\text{cosh}(\lambda_{1}x)+B\;\text{sinh}(\lambda_{1}x)+\frac{2r_{p}{ɛ}_{0}}{\pi r_{f}^{2}\lambda_{1}^{2}}\int_{0}^{{\text{cos}}^{-1}(\frac{b}{r_{p}})}\frac{1}{\frac{r_{p}(1-\text{sin }\theta )}{G_{a}}+\frac{r_{p}}{G_{c}}\text{ln}(\frac{r_{p}}{r_{f}})}d\theta$$

where the constants A and B can be determined by the boundary conditions at the ends of bonded region as follows:

(20) $$\sigma_{1}^{f}=0\;x=\pm L_{f}\;L_{f}:\text{half}\;\text{of}\;\text{the}\;\text{bonded}\;\text{length}$$

By enforcing the boundary conditions Equation (20), the stress of optical fiber Equation (19) can be obtained as:

(21) $$\sigma_{1}^{f}=\frac{{ɛ}_{0}}{\left(\frac{\pi r_{f}^{2}}{2hr_{p}E_{h}}+\frac{1}{E_{f}}\right)}[1-\frac{\text{cosh}(\lambda_{1}x)}{\text{cosh}(\lambda_{1}L_{f})}]$$

The strain in the optical fiber induced by the host material is:

(22) $${ɛ}_{1}^{f}=\frac{{ɛ}_{0}}{E_{f}(\frac{\pi r_{f}^{2}}{2hr_{p}E_{h}}+\frac{1}{E_{f}})}[1-\frac{\text{cosh}(\lambda_{1}x)}{\text{cosh}(\lambda_{1}L_{f})}]$$

where *ɛ*_0_ represents the strain applied on the host material.

## 3. Parametric Study

In this section, a parametric analysis was conducted using Equation (22) to investigate the influences of the bonded length and coating on the optical fiber strain. The material properties for host material, adhesive, coating and optical fiber used in the numerical study are listed in Table 1. The outer radii of optical fiber and coating are *r_f_* = 62.5 μm and *r_p_* = 125 μm, respectively; the thickness of the host material and the width of the gap are *h* = 8 mm and *b* = 0.2*r_p_*, respectively. The index of refraction and pockel’s constants [21] are *n*_0_ = 1.45, *p*_11_ = 0.12, *p*_12_ = 0.27, respectively.

**Figure 3.**
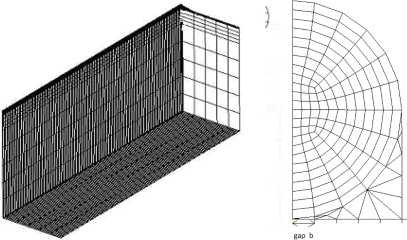
Finite element mesh.

**Figure 4.**
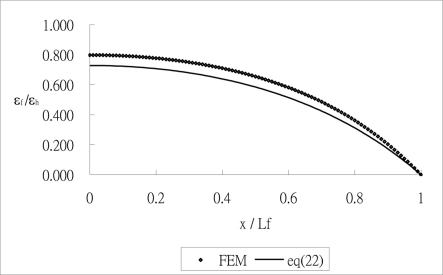
comparison of the normalized strain along the optical fiber obtained by FEM and Equation (22).

Prior to the parametric analysis, the theoretical prediction Equation (22) of the strain in the optical fiber is validated by the finite element method using the commercial software ANSYS. Eight node elements (solid 45) were used to generate meshes as shown in Figure 3. The strain of the optical fiber along the surface bonded length calculated by Equation (22) and finite element method are plotted in Figure 4. The optical fiber strain *ɛ_f_* shown in Figure 4 is normalized by the far field strain *ɛ*_0_ which is applied to the host material. It is shown that the theoretical predictions are in good agreement with the FEM results. The strain distribution of the optical fiber shows that the maximum strain occurs in the middle of the surface bonded optical fiber and decreases to zero at both ends of the bonded length.

### 3.1. Influence of the Surface Bonding Length

**Figure 5.**
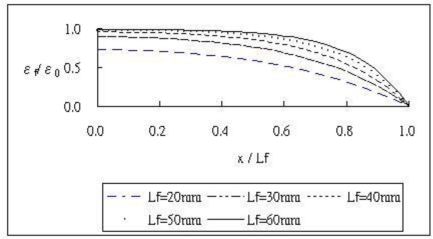
normalized strain along the optical fiber with different bonded length.

A variety of surface bonded lengths 2*L_f_* = 40 mm, 60 mm, 80 mm, 100 mm, 120 mm were used to study the influence of the bonded length on the optical fiber strain when the host material is subjected to a far field strain *ɛ*_0_. Figure 5 shows the results of normalized optical fiber strain *ɛ*_f_/*ɛ*_0_ along the bonded region calculated by Equation (22) with different bonded lengths. It clearly indicates that the longer of the bonded length the more strain is transferred to the optical fiber. A steady strain can be achieved in the middle of the optical fiber only when the bonded length is long enough. The strain in the optical fiber is less than the strain in the host structure due to the shear deformation at the coating and adhesive.

### 3.2. Influence of the Young’s Modulus of the Coating

**Figure 6.**
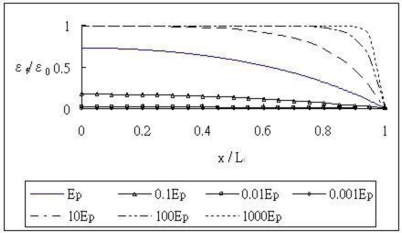
Normalized strain along the optical fiber with different modulus of the coating.

The host material, adhesive and optical fiber were the same as the previous example listed in Table 1. The Young’s modulus of the coating varied from 0.001 *E_p_* to 1,000 *E_p_* (*E_p_* = 6.7 MPa). Figure 6 shows the normalized strain of the optical fiber along the bonded length (2*L_f_* = 40 mm) with different modulus of the coating. From this figure, it is apparent that the strain transfer is increasing as the Young’s modulus of the coating is increasing. The results indicate that if a high modulus material is used as the coating, the interface bonding becomes more rigid. Thus, the strain transferred from the host to the optical fiber becomes more effective.

## 4. Mach-Zehnder Interferometric Sensor

**Figure 7.**
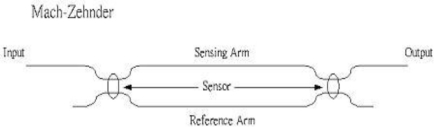
Mach-Zehnder interferometer.

Interferometric sensors have received much attention due to their high sensitivity and high spatial resolution. The Mach-Zehnder optical fiber sensor is perhaps the best known because it was developed first. This interferometer acts in the classic sense by optically interfering the light propagating in the reference and sensing fibers. A schematic diagram of a Mach-Zehnder interferometer is shown in Figure 7. It consists of two 2 × 2 couplers at the input and output. The excitation is applied to the sensing fiber, resulting optical path difference between the reference and sensing fibers.

The light intensity of the output of the Mach-Zehnder interferometer can be expressed as [22]:

(23) $$\begin{array}{l} I=2A^{2}(1+\text{cos}\;\Delta \varphi )\\ \Delta \varphi =\frac{2\pi n_{0}}{\lambda }\left\{1-\frac{n_{0}}{2}[(1-v)p_{12}-vp_{11}]\right\}\int_{\Gamma }{ɛ}_{1}^{f}dx \end{array}$$

where Δ*φ* is the optical phase shift, *n*_0_ is the refractive index of the optical fiber, *λ* is optical wavelength, *ν* is the Poisson’s ratio, *P*_11_ and *P*_12_ are the Pockel’s constants, ${ɛ}_{1}^{f}$ is the strain of the optical fiber. Since the terms in front of the integral sign of Δ*φ* are constants for any given optical fiber system, the total optical phase shift Δ*φ* is proportional to the integral of the optical fiber strain. Thus, by measuring the total optical phase shift, the integral of the optical fiber strain can be easily obtained as follows:

(24) $$\int_{\Gamma_{s}}{ɛ}_{1}^{f}dx=\frac{\Delta \varphi }{\frac{2\pi \;n_{0}}{\lambda }\left\{1-\frac{1}{2}n_{0}^{2}[(1-v_{f})p_{12}-v_{f}p_{11}]\right\}}$$

The light intensity shown in Equation (23) is a cosine function of optical phase shift. As the optical phase shift Δ*φ* = 2*π*, the light intensity goes through a complete cycle. The corresponding integral of the strain is:

(25) $$\Delta s=\int_{\Gamma_{s}}{ɛ}_{1}^{f}dx=\frac{2\pi }{\frac{2\pi \;n_{0}}{\lambda }\left\{1-\frac{1}{2}n_{0}^{2}[(1-v_{f})p_{12}-v_{f}p_{11}]\right\}}=\frac{\lambda }{n_{0}\left\{1-\frac{1}{2}n_{0}^{2}[(1-v_{f})p_{12}-v_{f}p_{11}]\right\}}$$

The integral of the strain in Equation (25) represents the total change of the length of the sensing fiber which is surface bonded on the host material. Thus, the average strain in the surface bonded optical fiber that induced one complete cycle of light intensity can be obtained:

(26) $$\Delta {ɛ}_{\mathrm{avg}}=\frac{\Delta s}{s}=\frac{\int_{\Gamma_{s}}{ɛ}_{1}^{f}dx}{2L_{f}}=\frac{\lambda }{2L_{f}n_{0}\left\{1-\frac{1}{2}n_{0}^{2}[(1-v_{f})p_{12}-v_{f}p_{11}]\right\}}$$

The total average strain in the surface bonded optical fiber can be calculated by counting the number of cycles of interferometric light intensity as follows:

(27) $${\left({ɛ}_{1}^{f}\right)}_{\text{exp}}=m\Delta {ɛ}_{\mathrm{avg}}=\frac{m\lambda }{2L_{f}n_{0}\left\{1-\frac{1}{2}n_{0}^{2}[(1-v_{f})p_{12}-v_{f}p_{11}]\right\}}$$

where m is the number of light intensity cycles counted from the experimental result, it can be integer or non-integer. Thus, the experimental result of the average strain of the surface bonded optical fiber using the Mach-Zehnder interferometric technique can be determined from Equation (27).

Theoretical prediction of the average strain of the surface bonded optical fiber can be deduced from Equation (22) as:

(28) $${\left({ɛ}_{1}^{f}\right)}_{\mathrm{the}}=\frac{\int_{\Gamma_{s}}{ɛ}_{1}^{f}(x)dx}{s}=\frac{\int_{-L_{f}}^{L_{f}}\frac{{ɛ}_{0}}{E_{f}(\frac{\pi r_{f}^{2}}{2hr_{p}E_{h}}+\frac{1}{E_{f}})}[1-\frac{\text{cosh}(\lambda_{1}x)}{\text{cosh}(\lambda_{1}L_{f})}]dx}{2L_{f}}$$

Define the coefficient of strain transformation between optical fiber and host material as follows:

(29) $${\left(K\right)}_{\text{exp}}=\frac{{\left({ɛ}_{1}^{f}\right)}_{\text{exp}}}{{ɛ}_{\mathrm{ave}}}=\frac{m\lambda }{2L_{f}n_{0}\left\{1-\frac{1}{2}n_{0}^{2}[(1-v_{f})p_{12}-v_{f}p_{11}]\right\}{ɛ}_{\mathrm{ave}}}$$

(30) $${\left(K\right)}_{\mathrm{the}}=\frac{{\left({ɛ}_{1}^{f}\right)}_{\mathrm{the}}}{{ɛ}_{\mathrm{ave}}}=\frac{\int_{-L_{f}}^{L_{f}}\frac{{ɛ}_{0}}{E_{f}(\frac{\pi r_{f}^{2}}{2hr_{p}E_{h}}+\frac{1}{E_{f}})}[1-\frac{\text{cosh}(\lambda_{1}x)}{\text{cosh}(\lambda_{1}L_{f})}]dx}{2L_{f}{ɛ}_{\mathrm{ave}}}$$

where *ɛ_ave_* denotes the average strain of the host material along the bonded length.

## 5. Experimental Test Results

**Figure 8.**
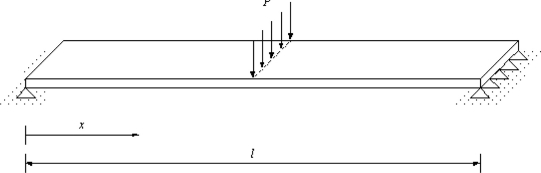
Test specimen.

**Figure 9.**
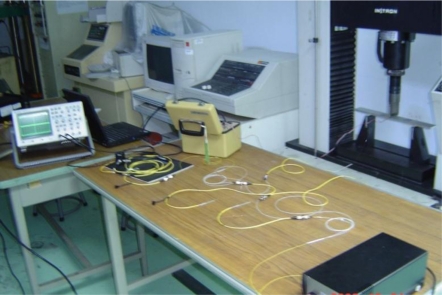
Experimental setup of the three point bending measured by Mach-Zehnder interferometry.

Three-point bending tests were conducted to examine the strain response of the optical fiber strain sensor. A test specimen made of aluminum is shown in Figure 8. An optical fiber was bonded on the bottom surface of the specimen in the central area as the sensing fiber of the Mach-Zehnder interferometer. Near the optical fiber, an electric resistance strain gauge with gauge length of 5 mm was adhered to the specimen to measure the strain *ɛ*_0_ of the host material and compared to the results from the optical fiber strain sensor. The material properties of the optical fiber and adhesive are shown in Table 1. The Young’s modulus and Poisson’s ratio for the host material (aluminum) are 72 GPa and 0.3, respectively. The wavelength *λ* of the light emitted from the laser diode to the optic fiber is 1,549.2 nm. The experimental setup is shown in Figure 9.

For the specimen subjected to a three-point bending test as shown in Figure 8, a linear variation of the strain is developed in the specimen as follows:

(31) $$\{\begin{array}{ll} {ɛ}_{0}=\frac{\mathrm{My}}{\mathrm{EI}}=\frac{\mathrm{Pxh}}{4\mathrm{EI}} & 0\leq x\leq \frac{l}{2}\\ {ɛ}_{0}=\frac{\mathrm{My}}{\mathrm{EI}}=\frac{\mathrm{Ph}}{4\mathrm{EI}}(l-x) & \frac{l}{2}\leq x\leq l \end{array}$$

where *P*, *l* and *I* are the applied load, length and moment of inertia of the specimen, respectively.

The average strain of the specimen along the bonded length can be calculated:

(32) $${ɛ}_{\mathrm{ave}}=\frac{\int_{-L_{f}}^{0}\frac{Pxh}{4E_{h}I}dx+\int_{0}^{L_{f}}\frac{Ph}{4E_{h}I}(l-x)dx}{2L_{f}}$$

**Figure 10.**
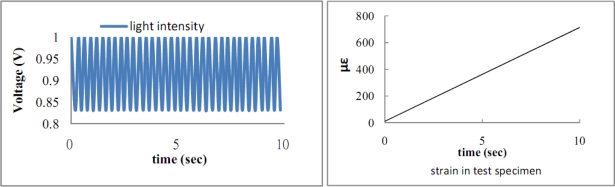
Light intensity of Mach-Zehnder interferometer and strain of test specimen measured by strain gauge for polymer coated optical fiber with bonded length 9 cm.

**Figure 11.**
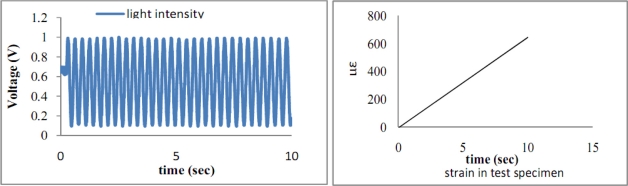
Light intensity of Mach-Zehnder interferometer and strain of test specimen measured by strain gauge for acrylate coated optical fiber with bonded length 9 cm.

The length of the surface bonded optical fiber is varied from 2*L_f_* = 40 mm to 120 mm, with increments of 10 mm in this experimental test to investigate the effect of the bonded length on the optical fiber strain. Optical fibers coated with two different materials polymer and acrylate were fabricated to evaluate the influence of the coating on the optical fiber strain. Polymer is the original coating of the optical fiber with Young’s modulus of 6.7 MPa [20]. Acrylate is the recoating of the optical fiber with Young’s modulus of 28 MPa provided by the manufacturer. Optical fiber without coating is also included as reference. Figure 10 shows the results of the strain in the test specimen measured by the strain gauge and the light intensity of the Mach-Zehnder interferometer for polymer coated optical fiber with bonded length of 90 mm. In the case of acrylate coating, the experimental results are shown in Figure 11.

It appears that the number of light intensity cycles increases with the increase in coating shear modulus, while the loading conditions including the end loads and loading rate remain the same. Counting the number of light intensity cycles from Figures 10 and 11 and then substituting into Equation (27) yields the experimental result of the average strain of the optical fiber. Theoretical prediction of the average optical fiber strain is obtained by substituting the strain *ɛ*_0_ of the test specimen from Equation (31) into Equation (28). Tables 2 and 3 show the results of the average optical fiber strain for various bonded lengths attained by experimental measurement and theoretical prediction with polymer and acrylate coating, respectively. Good agreement is achieved between the theoretical calculation Equation (28) and the experimental measurement [Equation (27)] with a difference of less than 10%. The strain transferred from the host material to the optical fiber in the case of optical fiber without coating is listed in Table 4.

**Figure 12.**
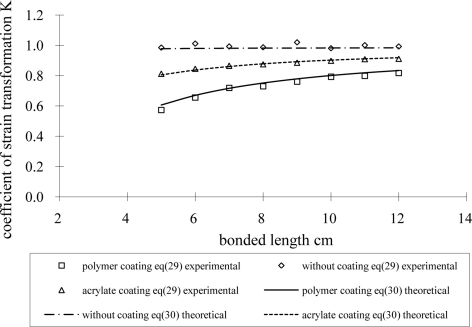
coefficient of strain transformation versus bonded length for polymer and acrylate coating obtained by experimental measurement Equation (29) and theoretical prediction Equation (30).

The coefficient of strain transformation between the optical fiber and host material can be obtained by utilizing Equation (29) for experimental measurement and Equation (30) for theoretical prediction. Figure 12 shows the results of the coefficient of strain transformation *versus* the bonded length for polymer and acrylate coatings. The coefficient of strain transformation for the optical fiber without coating is also presented in Figure 12. From Table 2, Table 3 and Figure 12, the optical fiber strain is not the same as that in the host material. The longer the bonded length the larger the coefficient of strain transformation, *i.e.*, the more strain is transferred to the optical fiber. The coefficient of strain transformation increases with the increase in coating shear modulus. The coefficient of strain transformation for optical fibers without coating shown in Figure 12 is close to 1, which means the strain in the host material can completely transfer to the optical fiber. However, optical fibers made from silicon dioxide are very fragile. They are easily broken, even when subjected to slight bending, so a polymer coating is required to protect the optical fiber from brittle fracture. The influence of the coating on the strain transformation rate between the optical fiber and surrounding host material is investigated experimentally in this section. The experimental results for the general strain transformation trend related to the bonded length and coating agree well with the theoretical predictions.

## 6. Conclusions

Smart structures using optical fiber as a strain sensor have become more attractive in recent years. Due to the shear deformation in the protective coating, the optical fiber strain is not exactly the same as that for the surrounding host material. Thus, the strain measured by the optical fiber needs to be modified to reflect the influence of the coating, to consequently improve the accuracy of the measurements. In this work, an analytical solution of the strain of surface bonded optical fiber induced by the host material to characterize the influence of the coating and bonded length is derived based on the theory of elasticity. Experimental measurements of the optical fiber strain were conducted using the Mach-Zehnder interferometric technique, and compared with the theoretical predictions. Good agreement was observed between the experimental measurements and theoretical prediction. The percentage of strain in the host material actually transferred to the optical fiber is dependent on the bonded length of the optical fiber and the protective coating. Parametric studies showed that the longer the bonded length and the stiffer the coating, the more strain is transferred to the optical fiber.

## Figures and Tables

**Table 1.** Material properties.

	**Host material**	**Adhesive**	**Coating**	**Optical fiber**
Young’s modulus(GPa)	72	2	0.0067	72
Poisson’s ratio	0.3	0.4	0.49	0.17

**Table 2.** Average strain of the polymer coated optical fiber obtained by the experimental measurement Equation (27) and theoretical calculation Equation (28) with different bonded length.

**Bonded Length (2*L_f_*)**	**Host material strain measured by the strain gauge (*μɛ*)**	**Applied load P (N)**	**Number of cycles**	**Experimental measurement Equation (27) of optical fiber strain *ɛ_f_* (*μɛ*)**	**Theoretical calculation Equation (28) of optical fiber strain *ɛ_f_* (*μɛ*)**	**Difference between Equation (27) and Equation (28) (%)**

5 cm	657	402	13.5	342	363	6.12
6 cm	655	397	15	374	387	3.5
7 cm	715	436	24	433	444	2.54
8 cm	641	387	23.8	374	396	5.74
9 cm	742	453	32.7	458	478	4.26
10 cm	675	402	33.2	419	425	1.43
11 cm	713	426	39	446	449	0.52
12 cm	676	407	41.3	434	424	2.26

**Table 3.** Average strain of the acrylate coated optical fiber obtained by the experimental measurement Equation (27) and theoretical calculation Equation (28) with different bonded length.

**Bonded Length (2*L_f_*)**	**Host material strain measured by the strain gauge (*μɛ*)**	**Applied load P (N)**	**Number of cycles**	**Experimental measurement Equation (27) of optical fiber strain *ɛ_f_* (*μɛ*)**	**Theoretical calculation Equation (28) of optical fiber strain *ɛ_f_* (*μɛ*)**	**Difference between Equation (27) and Equation (28) (%)**

5 cm	686	407	16.8	502	507	1.08
6 cm	698	414	20.9	519	527	1.48
7 cm	679	402	23.6	503	513	1.93
8 cm	669	397	25.9	483	494	2.45
9 cm	706	419	28.9	479	527	10.18
10 cm	703	419	33.6	502	520	3.65
11 cm	687	409	35.5	482	499	3.66
12 cm	670	397	35	435	475	9.12

**Table 4.** average strain of the optical fiber without coating obtained by the experimental measurement Equation (27) and theoretical calculation Equation (28) for different bonded length.

**Bonded Length (2*L_f_*)**	**Host material strain measured by the strain gauge (*μɛ*)**	**Applied load P (N)**	**Number of cycles**	**Experimental measurement Equation (27) of optical fiber strain *ɛ_f_* (*μɛ*)**	**Theoretical calculation Equation (28) of optical fiber strain *ɛ_f_* (*μɛ*)**	**Difference between Equation (27) and Equation (28) (%)**

5 cm	664	409	19.5	582	620	6.16
6 cm	674	414	23.9	594	616	3.63
7 cm	654	387	30.9	557	563	1.08
8 cm	672	399	29.1	543	556	2.66
9 cm	746	443	35.3	584	615	4.97
10 cm	737	443	38.4	572	600	4.49
11 cm	672	409	38.3	520	538	3.47
12 cm	666	397	39.8	494	508	2.78
